# Clustered PHD domains in KMT2/MLL proteins are attracted by H3K4me3 and H3 acetylation-rich active promoters and enhancers

**DOI:** 10.1007/s00018-022-04651-1

**Published:** 2023-01-04

**Authors:** Anna Maria Stroynowska-Czerwinska, Magdalena Klimczak, Michal Pastor, Asgar Abbas Kazrani, Katarzyna Misztal, Matthias Bochtler

**Affiliations:** 1 International Institute of Molecular and Cell Biology, 02-109 Warsaw, Poland; 2 Institute of Biochemistry and Biophysics, Polish Academy of Sciences, 02-106 Warsaw, Poland; 3 Institut de Génétique et de Biologie Moléculaire et Cellulaire, Illkirch-Graffenstaden, France

**Keywords:** Chromatin reader domain, Multiple PHD domains, PHD domain readout, Tandem domain, Histone mark cross-talk, Cancer

## Abstract

Histone lysine-specific methyltransferase 2 (KMT2A-D) proteins, alternatively called mixed lineage leukemia (MLL1-4) proteins, mediate positive transcriptional memory. Acting as the catalytic subunits of human COMPASS-like complexes, KMT2A-D methylate H3K4 at promoters and enhancers. KMT2A-D contain understudied highly conserved triplets and a quartet of plant homeodomains (PHDs). Here, we show that all clustered (multiple) PHDs localize to the well-defined loci of H3K4me3 and H3 acetylation-rich active promoters and enhancers. Surprisingly, we observe little difference in binding pattern between PHDs from promoter-specific KMT2A-B and enhancer-specific KMT2C-D. Fusion of the KMT2A CXXC domain to the PHDs drastically enhances their preference for promoters over enhancers. Hence, the presence of CXXC domains in KMT2A-B, but not KMT2C-D, may explain the promoter/enhancer preferences of the full-length proteins. Importantly, targets of PHDs overlap with KMT2A targets and are enriched in genes involved in the cancer pathways. We also observe that PHDs of KMT2A-D are mutated in cancer, especially within conserved folding motifs (Cys4HisCys2Cys/His). The mutations cause a domain loss-of-function. Taken together, our data suggest that PHDs of KMT2A-D guide the full-length proteins to active promoters and enhancers, and thus play a role in positive transcriptional memory.

**Graphical Abstract:**

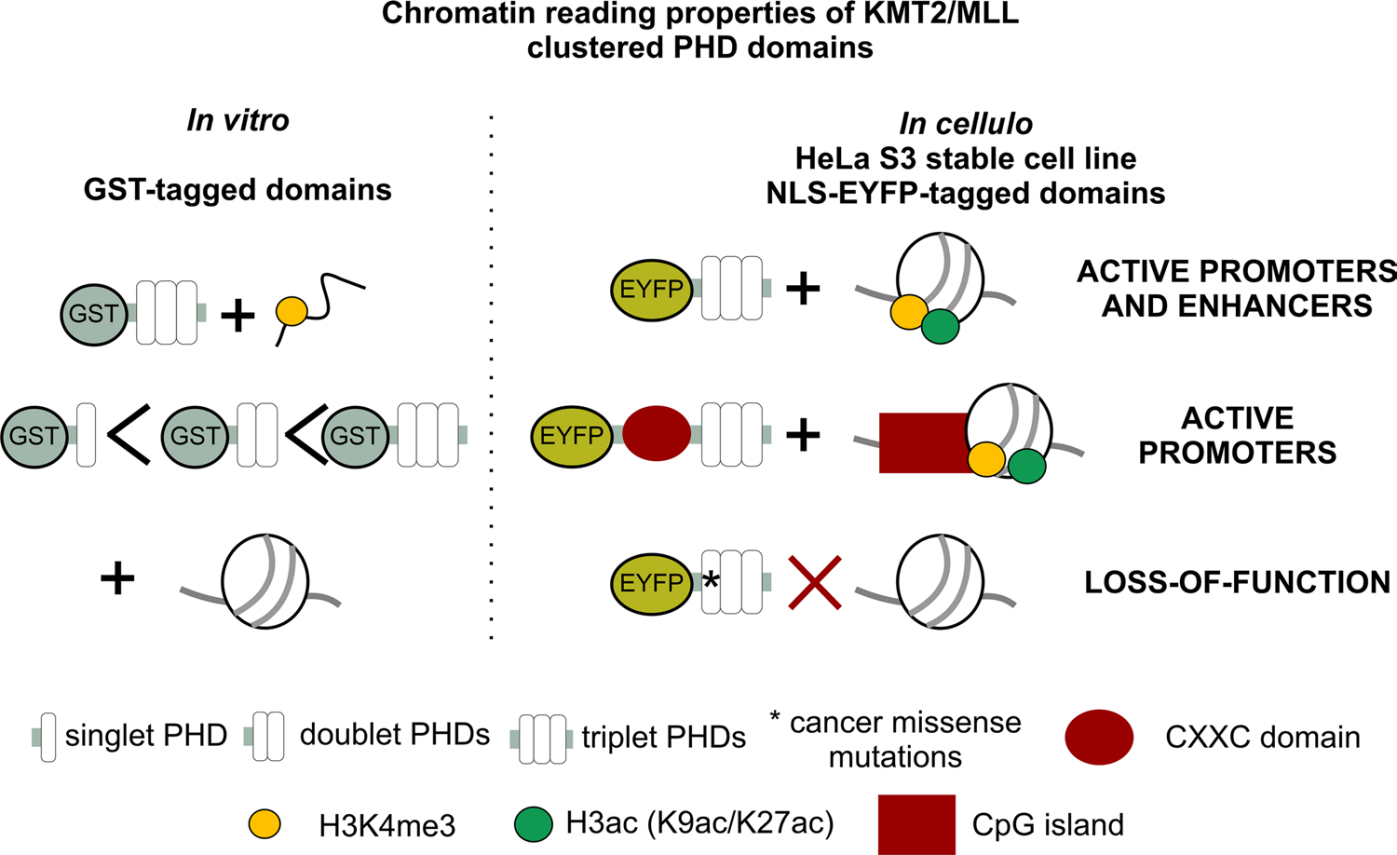

**Supplementary Information:**

The online version contains supplementary material available at 10.1007/s00018-022-04651-1.

## Introduction

Histone lysine-specific methyltransferases KMT2A-D are catalytic engines of human COMPASS-like (hCOMPASS-like) complexes that mediate positive transcriptional memory, by depositing the activating mark H3K4me1-3 at regulatory elements (promoters and enhancers) of already active genes [1–4]. hCOMPASS-like complexes share many subunits, including WD repeat-containing protein 5 (WDR5), Retinoblastoma-binding protein 5 (RbBP5), Absent small homeotic-2 like (ASH2L), and Dumpy-30 (DPY-30), that together form the shared WRAD sub-complex [5]. KMT2A-D are alternatively termed mixed lineage leukemia proteins (MLL1-4), due to KMT2A/MLL1 originally being identified as a frequent breakpoint in mixed lineage leukemia (MLL) [6]. Mutations of KMT2C/MLL3 and KMT2D/MLL4 genes are also found in other malignancies [7, 8]. The KMT2C/MLL3 gene is frequently mutated and listed among pan-cancer drivers [9–11].

hCOMPASS-like complexes containing KMT2A-B are recruited to active promoters, where they deposit the active promoter modification H3K4me3 [4, 12–14]. In contrast, the hCOMPASS-like complexes with KMT2C or KMT2D preferentially bind to enhancers, where they deposit the enhancer mark H3K4me1 [15–17]. The complex chromatin targeting is influenced by both non-catalytic subunits [14, 18, 19] and intrinsic preferences of the KMT2A-D proteins [20]. However, the mechanistic causes for the promoter or enhancer preference are not well understood.

**Fig. 1:**
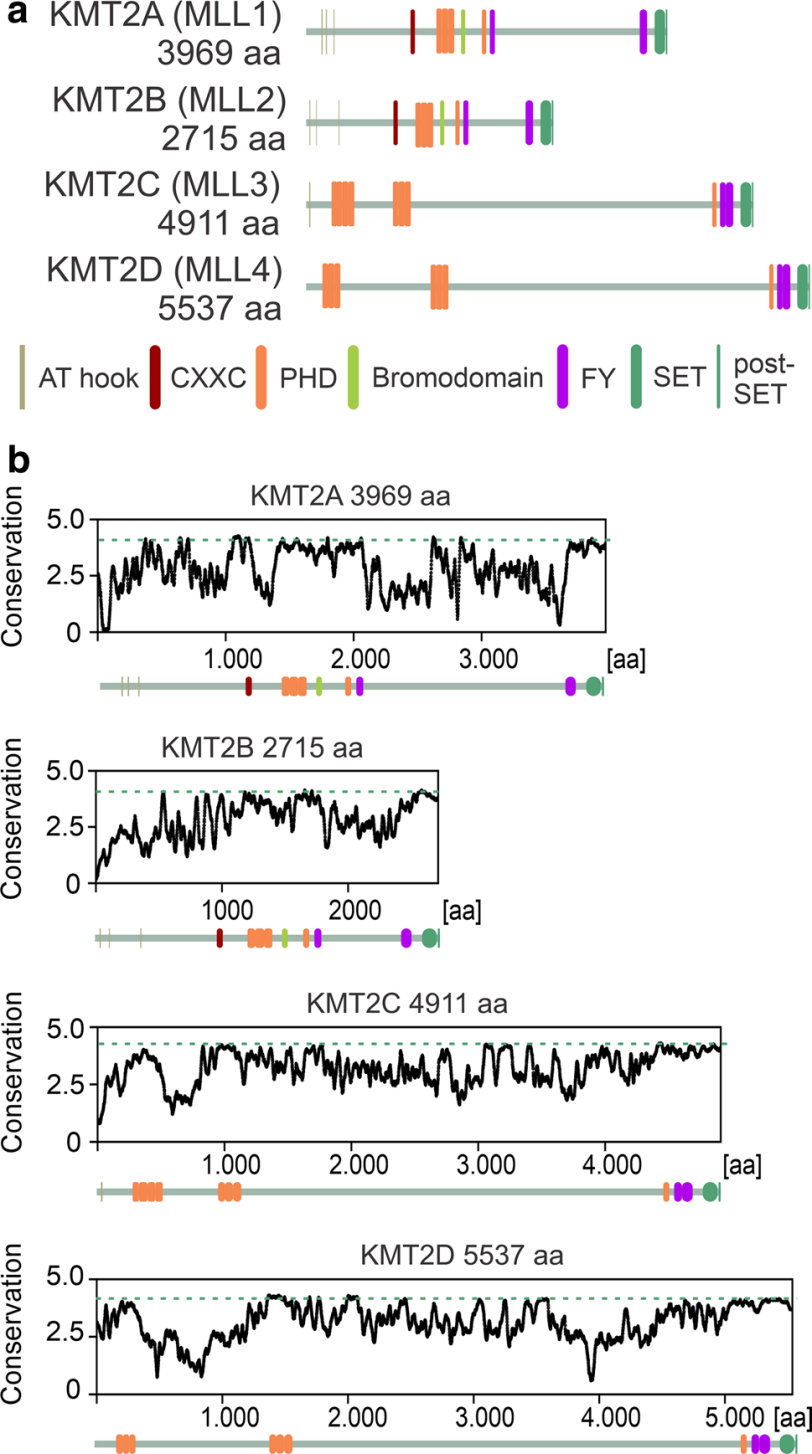
Clustered PHD domains (PHDs) are highly conserved in KMT2A-D proteins. **a** The architecture of KMT2A-D proteins. **b** Conservation of KMT2A-D proteins across species, measured in bits of information as in sequence logos. Domain architecture is indicated below the graphs. All diagrams are on the same scale. The dashed line shows the extent of conservation of the SET domain

The domain organization of the KMT2A-D proteins provides some clues about their binding preferences (Fig. 1a). Only promoter-specific KMT2A and KMT2B possess CXXC domains which bind non-modified CpGs [21–23], the hallmark of active promoters [24]. Furthermore, all KMT2A-D proteins contain multiple chromatin readers - the PHD domains (PHDs). Stand-alone PHDs are widely present in transcription-regulating proteins from plants to animals, and even in yeast [25]. These compact domains consist of around 65 amino acids and contain a Cys4HisCys2Cys/His (C4HC2C/H) folding motif, which chelates two structural Zn^2+^ ions [25]. PHD domains are known primarily as readers of H3K4me3 [26, 27], which they accommodate in a deep, hydrophobic pocket that is present in many PHD domains [28]. In addition, rare double PHD domains in MOZ and DPF bind acetylated lysine (H3K14ac) also in a deep, hydrophobic pocket, which is distinct from the methyl binding pocket [29, 30].

In KMT2A-D, most PHDs are arranged in clusters of multiple domains as triplets or even a quartet. This arrangement is unique in the human proteome but has received only little attention. Prior studies of PHD domains in KMT2A-D have typically focused on single PHD domains and a set of candidate chromatin marks as potential binding partners (H3K4me3, non-modified H4, H4R3me2, H4K16ac, H4K20ac and H4K20me1/3) [28, 31–33]. Surprisingly, H4K16ac has been found to be bound by PHD6 of KMT2D outside the deep acetylation pocket, in a fairly shallow region of the protein surface [31].

In this research, we aimed to understand the promoter and enhancer specificity of KMT2A-D proteins by characterizing PHD domains for their chromatin-binding properties, specifically in the context of the cellular chromatin environment. We present the first unbiased genome-wide characterization of the cellular binding sites of clustered PHDs in KMT2A, KMT2C and KMT2D proteins. We show that all clustered PHDs bind preferentially to well-defined loci in H3K4me3 and acetylation-rich active promoters and enhancers (especially H3K9ac and H3K27ac) of cancer-related genes. The loci bound by PHDs overlap partially but significantly with targets of full-length hCOMPASS-like subunits (KMT2A, WDR5 and RbBP5). Fusion of the CXXC domain to the clustered PHDs of KMT2A increases its affinity to promoters and improves the recapitulation of binding properties of the full-length protein. Our experimental data and the frequent loss-of-function cancer mutations in KMT2A-D PHD domains point to an important role of PHD domains in targeting hCOMPASS-like complexes to active promoters and enhancers.

## Results

### PHDs are highly conserved in KMT2A-D proteins

Amino acid conservation between PHDs of some mammals is surprisingly high (~ 99% identity) and prompted us to analyze the conservation of orthologues from a broader range of species (Suppl. Figure 1a). This comparison indicated that PHDs are among the most conserved regions within KMT2A-D proteins (Fig. 1b), in some cases (i.e., PHD4-6 of KMT2D) rivaling conservation of the catalytic SET domains. Differences between paralogous clustered PHDs in human KMT2A-D proteins are considerable, with typical values for amino acid identity and similarity below 40% and 50%, respectively (Suppl. Figure 1b). The high conservation between species and variability among different modules suggests that PHDs play an important role in KMT2A-D biology.

### Interaction between PHDs and histones strengthens with an increasing number of PHDs

**Fig. 2:**
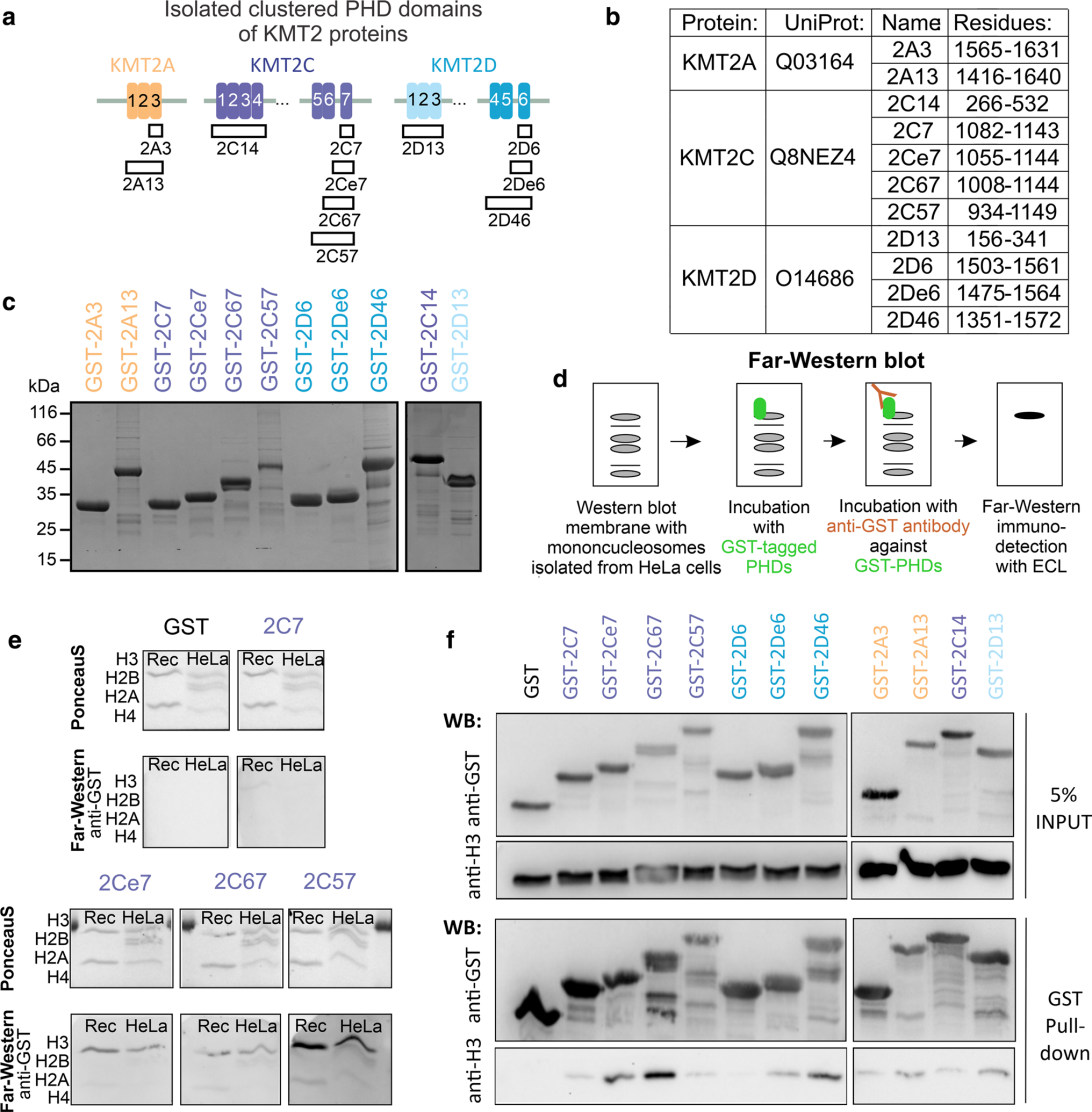
The affinity between PHDs and histones grows with the number of PHDs. **a** Overview of all purified GST-tagged PHDs. **b** Table with construct labels and protein regions, based on the UniProt entries. **c** 12% SDS-PAGE gel with 140 pmol purified isolated PHD domains stained with Coomasie blue. **d** Scheme of Far-Western blot methodology. **e** Far-Western blot performed with 100 nM solution of singlets (2C7, 2Ce7), doublet (2C67), and triplet (2C57) PHDs with a membrane containing denatured recombinant H3/H4 (Rec) and endogenous histones isolated from HeLa S3 cells. All membranes were exposed simultaneously. **f** Western blot of the GST pull-down samples with singlets (2A3, 2C7, 2D6), enhanced singlets (2Ce7, 2De6), doublet (2C67), and full-length clusters (2A13, 2C14, 2C57, 2D13, 2D46) of GST-tagged PHDs. Bound mononucleosomes were identified using an anti-H3 antibody. The experiments were performed at the same time, at least with two replicates

For in vitro biochemical characterisation, we cloned and purified from *E. coli* bacterial lysates all clustered PHDs of KMT2A-D proteins as well as some singlets or doublets as fusion proteins with GST-tag (Fig. 2a-c). We excluded PHDs of KMT2B from further studies, as they were poorly expressed and had low solubility. Hereafter, we designate individual PHDs by their paralogue identifier (2A-D) and by their position, i.e., the third PHD in KMT2A is abbreviated as 2A3. Similarly, clusters of PHDs are designated by the first and last domains, e.g., 2A13 comprises PHD1, PHD2 and PHD3 of KMT2A. PHDs containing an extension with the C4 zinc finger are termed “extended” PHDs [32] and are abbreviated with an extra “e”: 2Ae3, 2Be3, 2Ce4, 2Ce7, and 2De6.

To characterize binding properties of PHD domains in vitro but within the nucleosome context, we used recombinant histones and endogenous mononucleosomes isolated from a HeLa S3 cell line (Suppl. Figure 2a). First, we investigated the in vitro interactions with denatured proteins on the membrane by Far-Western blot (Fig. 2d-e, Suppl. Figure 2b). The analysis indicated that all GST-PHDs, but not GST alone, bound to both recombinant and endogenous H3, whereas other histones were bound either weakly or not at all. The strength of the histone interactions grew with the number of PHDs (e.g., 2C7 < 2Ce7 < 2C67 < 2C57) (Fig. 2e). For the triplets and the quartet, the interaction was strongest for 2D46 and 2C57 and weaker for 2A13, 2C14, and 2D13 (Suppl. Figure 2b). Histone modifications did not have a clear impact on the binding of PHDs, as both recombinant unmodified and endogenous modified H3 histones were bound with comparable affinity.

Next, we tested the interaction of PHDs with endogenous mononucleosomes in solution and performed GST pull-down assays (Fig. 2f). Consistent with the Far-Western results, we observed stronger interactions with PHD doublets and triplets than with singlets. In the Far-Western assay, the 2C57 triplet bound mononucleosomes better than the 2C67 doublet, but the opposite was observed in the pull-down assay. Possibly this discrepancy is a consequence of the lower stability of 2C57 during the longer overnight incubation used for the pull-down experiments. These results suggest that collaboration between the clustered PHD modules in KMT2A-D strengthens histone interactions.

### KMT2A-D clustered PHDs recognize specifically a subset of active promoters and enhancers

**Fig. 3:**
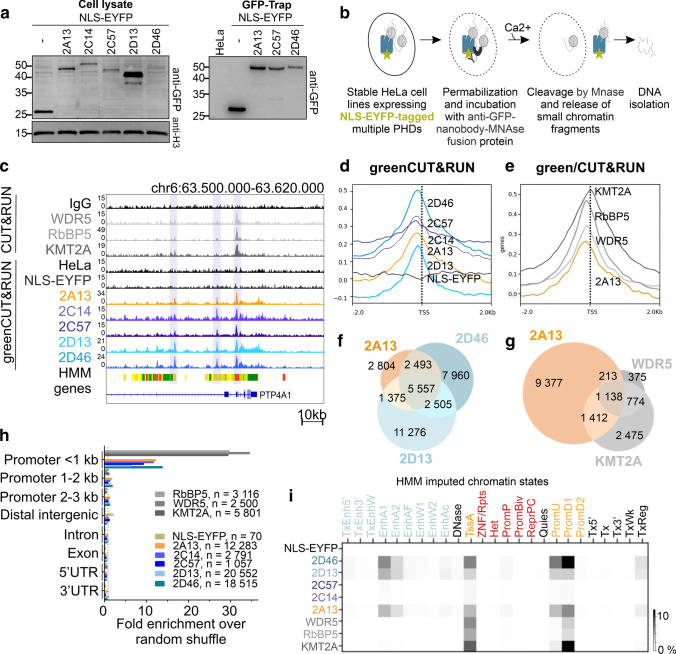
PHDs, expressed in the HeLa S3 cell lines, recognize specifically a subset of active promoters and to a lesser extent enhancers. **a** Western blot with anti-GFP antibody to detect NLS-EYFP-tagged clustered PHDs domains, expressed in stable HeLa S3 cell lines, generated by lentiviral transduction. Left panel—whole cellular lysate. Right panel—enriched proteins after GFP-Trap. **b** Scheme of greenCUT&RUN (gC&R) experiments.** c** IGV genome browser, showing read patterns of C&R hCOMPASS-like core subunits (WDR5, RbBP5 and KMT2A) and gC&R of clustered PHDs (2A13, 2C14, 2C57, 2D13, 2D46). The HMM lane shows imputed chromatin states; active promoter regions in red, active enhancers in yellow and orange and transcribed regions in green. **d-e** Heatmap plots comparing the distribution of reads around the TSS region (± 2 kb) of **d** clustered PHDs and NLS-EYFP (negative control) gC&R signals in comparison with the background of parental HeLa S3 cells and **e** hCOMPASS-like core subunits in comparison with IgG negative control. **f-g** Venn diagrams comparing MACS2 peaks of **f** selected PHDs (2A13, 2D13, 2D46) and **g** 2A13 with full-length hCOMPASS-like subunits (KMT2A, WDR5). Randomly shuffled data are shown in Suppl. Figure 3h. **h** Genomic distribution of PHDs and selected hCOMPASS-like subunits peaks. The quantification shows the enrichment in comparison with a random shuffle. Distribution without enrichment is shown in Suppl. Figure 3j. **i** Jaccard analysis comparing the genome-wide distribution of peaks of selected proteins and HMM imputed chromatin states. Enhancers are in light blue, promoters in yellow, poised or repressed chromatin states in red, and other chromatin states in black. The darker the signal, the stronger overlap is observed between the two samples. A detailed characterisation of imputed chromatin promoter and enhancer states considering chromatin marks is shown in Suppl. Figure 5c

As our previous observations showed that full-length clustered PHD domains are stronger chromatin binders than their shorter fragments, we aimed to examine them in the cellular chromatin environment. Therefore, we generated stable HeLa S3 cell lines expressing NLS-EYFP-tagged triplet and quartet PHDs (NLS-EYFP-2A13, NLS-EYFP-2C14, NLS-EYFP-2C57, NLS-EYFP-2D13, and NLS-EYFP-2D46), as well as NLS-EYFP alone, as a negative control. In general, the recombinant proteins were weakly expressed (predominantly 2D46) (Fig. 3a, Suppl. Figure 3a-c). Despite cell sorting after viral infection, the expression varied between cell lines and was anti-correlated with the strength of the interaction with mononucleosomes in Far-Western and GST pull-down (Fig. 2e, f), i.e., the strongest binder 2D46 was the least expressed, and 2D13 as one of the weakest binders was most highly expressed. We suspect that binding of the PHD domains to important chromatin regions affected the expression level and cells adjusted it to tolerable levels. The EYFP fluorescence signal in the nucleus was diffuse, excluding protein overexpression artifacts from aggregation (Suppl. Figure 3d). The immunofluorescence pattern of endogenous full-length KMT2D protein did not show a punctate signal distribution either (Suppl. Figure 3d).

To gain genome-wide insight into chromatin reader interaction patterns, we used stable cell lines for greenCUT&RUN (gC&R). gC&R relies on an MNase-anti-GFP nanobody, which cuts DNA around nucleosomes that are bound by an EYFP-tagged fusion protein of interest (Fig. 3b). For a better understanding of the role of PHD domains in the recruitment of KMT2A-D proteins, we also used standard C&R with antibodies against full-length KMT2 (KMT2A, KMT2C and KMT2D) proteins and hCOMPASS-like subunits (WDR5, RbBP5). The antibodies against full-length KMT2C and KMT2D did not yield useful sequencing data.

Read patterns of the gC&R data appeared similar for all clustered PHDs when inspected in a genome browser (Fig. 3c). The NLS-EYFP negative control was comparable to the parental HeLa S3 background, indicating that the NLS-EYFP-tag did not cause artificial chromatin binding (Fig. 3c). Moreover, binding sites for PHDs and full-length proteins typically overlapped (Fig. 3c). Genomic heatmaps indicated strong enrichment of PHDs around transcriptional start sites (TSS), with the peak just upstream (~ 150-200nt) (Fig. 3d). We also observed a similar distribution for the investigated full-length hCOMPASS-like subunits. However, the sharp peak of KMT2A was shifted and located exactly at the TSS (Fig. 3e). The nearly symmetrical peaks extended into the promoter region, but also downstream into the transcribed region.

We obtained good-quality sequencing data for NLS-EYFP-2A13 (~ 12 000 peaks), NLS-EYFP-2D13 (~ 20 500 peaks), NLS-EYFP-2D46 (~ 18 500 peaks) and moderate-quality sequencing data for NLS-EYFP-2C14 (~ 2 800 peaks) and NLS-EYFP-2C57 (~ 1 100 peaks). Importantly, the negative control of NLS-EYFP showed only around 70 peaks. In agreement with the observation from inspection in a genome browser, many binding sites of the PHDs were shared (~ 5 500 peaks for NLS-EYFP-2A13, NLS-EYFP-2D13, and NLS-EYFP-2D46) for the actual, but not randomly shuffled data (Fig. 3f, Suppl. Figure 3e, f). For the full-length proteins, we observed very well-defined, but fewer (2 500—5 800) binding sites than for the PHDs. These binding sites strongly overlapped with each other (~ 1 200 common peaks, Suppl. Figure 3g) and partially with clustered PHDs (Fig. 3g, Suppl. Figure 3h ~ 1 100 common peaks for NLS-EYFP-2A13, KMT2A, and WDR5, Suppl. Figure 3i ~ 800 common peaks for NLS-EYFP-2A13, 2D13, 2D46, KMT2A, and WDR5). The overlap was absent upon random shuffle, confirming its relevance (Fig. 3g, Suppl. Figure 3h).

Compared to randomly shuffled data, PHD domains and full-length proteins were 10- to 15-fold and 30- to 35-fold more enriched near promoters (< 1 kb), respectively (Fig. 3h, Suppl. Figure 3j). To classify these binding sites more precisely, we assigned them to imputed chromatin states for HeLa S3 cells according to a “25 chromatin states” hidden Markov model (HMM) [34]. Overlap between genomic regions was quantified using the Jaccard index, defined as the ratio of the intersection and the union of two regions of interest. This analysis showed that all PHDs were bound at a subset of active promoters (active TSS (TssA), promoter downstream TSS 1 (PromD1), promoter upstream TSS (PromU), but not promoter downstream TSS 2 (PromD2)) and to a lesser extent at a subset of active and strong enhancers (EnhA1, EnhA2) (Fig. 3i, Suppl. Figure 3k). In contrast, the full-length subunits mapped exclusively to active promoters (mostly TssA and PromD1) (Fig. 3i). Taken together, these findings support the hypothesis that clustered PHDs play an important role in the targeting of the hCOMPASS-like complexes to active regulatory elements. Contrary to our initial expectation, all PHDs, also from enhancer-specific KMT2C-D proteins, are similarly enriched mostly at promoters and to a lesser extent at enhancers.

### Clustered PHDs bind H3K4me3 and H3 acetylation-rich loci, especially containing H3K9ac and H3K27ac, but not H4K16ac

To better characterize loci bound by PHDs, we used a comprehensive set of HMM-imputed chromatin marks [34]. First, we validated this set with experimental ChIP-Seq data from the ENCODE database [35], and our CUT&RUN data for H3K4me1 or H3K4me3 in HeLa S3 cells (Suppl. Figure 4a, b). Next, we quantified the overlap between chromatin regions bound by PHD domains and HMM imputed histone marks, using the Jaccard overlap measure (Suppl. Figure 4c, d). The analysis showed that clustered PHDs were specifically recruited to regions enriched with the active promoter and enhancer marks (H3K4me1-3/ac, H3K9ac, and H3K27ac), but not to poised or silent chromatin marks, like H3K27me3 or H3K9me3 (Suppl. Figure 4c, d). This pattern is consistent with bound imputed chromatin states (Fig. 3i). We also observed a strong correlation of PHD domain binding sites with other acetylation marks, like H2BK15ac, H3K23ac, and to a lesser extent H4 acetylation (H4K5ac, H4K8ac, H4K12ac) (Suppl. Figure 4c, d). No such preference was detected for the NLS-EYFP negative control.

**Fig. 4:**
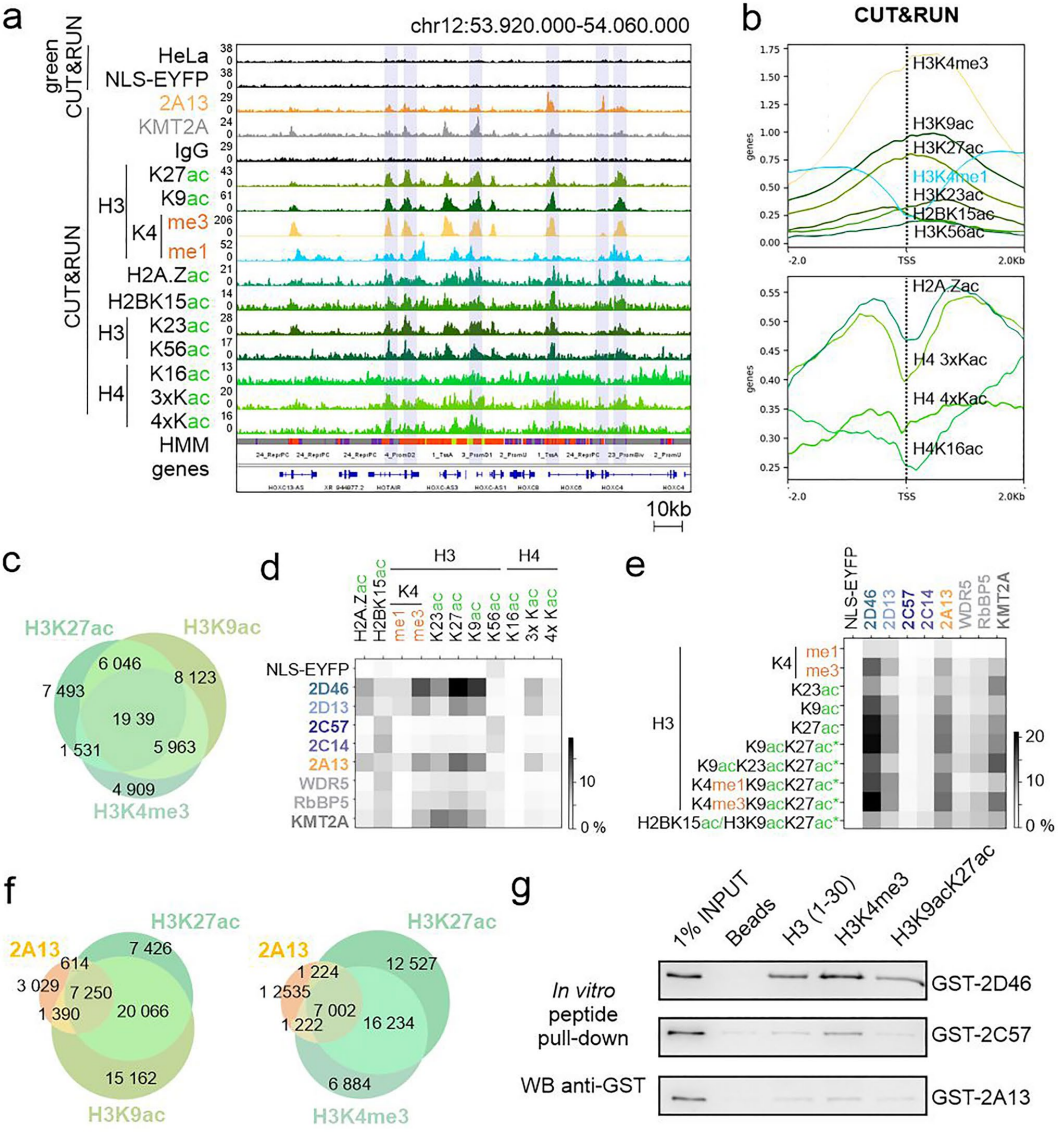
PHDs recognize loci enriched with multiple acetylation at H3 and H3K4me3, but not H4K16ac. **a** IGV genome browser snapshots of the distribution of the reads within the HOXC cluster of 2A13, NLS-EYFP gC&R, full-length KMT2A and selected histone marks (based on HMM imputed chromatin marks, Suppl. Figure 4c): H3K4me1/3, H3K9ac, H2K23ac, H3K27ac, H3K56ac, H2A.Zac, H2BK15ac, H4K16ac, H4K16ac, H43xKac (H4K5acK8acK12ac) and H44xKac (H4K5acK8acK12acK16ac) C&R data. **b** Heatmap plots around the TSS region (± 2 kb) of analyzed histone marks in comparison with IgG negative control. Upper panel—H2B and H3 modifications. Bottom panel—H2A.Z and H4 modifications. **c** Venn diagram comparing peaks of the H3K4me3, H3K9ac and H3K27ac. **d** Jaccard analysis comparing the genome-wide peak distribution of PHDs or hCOMPASS-like subunits and selected histone marks as well as **e** intersections (labeled with asterisk “*”) of the strongest correlated marks (H3K4me1/3, H3K9ac, H3K23ac, H3K27ac). **f** Venn diagrams comparing the distribution of peaks. Left panel—2A13 PHDs,H3K9ac and H3K27ac. Right panel—2A13, H3K27ac and H3K4me3. **g** Western blot with anti-GST antibody after biotinylated peptide in vitro pull-down with GST-tagged PHDs expressed in *E. coli*. Beads without peptides served as a negative control

To confirm the strongest correlations of PHD binding sites determined by gC&R with imputed chromatin states, we selected antibodies against H3K4me1/3, H3K9ac, H3K23ac, H3K27ac, H3K56ac and H2BK15ac for the standard CUT&RUN. We also included H4K16ac, which was previously reported as a 2D6 target [25], as well as antibodies raised against multiple acetylated H2A.Zac and H4 histone peptides (H4K5acK8acK12ac (H4 3xKac) and H4K5acK8acK12acK16ac (H4 4xKac)). We discovered that most acetylation marks were broadly distributed within actively transcribed gene bodies (Fig. 4a) with the exceptions of H3K9ac and H3K27ac, which were much more specific. Genomic heatmap plots indicated that H3 and H2B acetylation marks and H3K4me3 signals peaked around the TSS, whereas H4 and H2A.Z acetylation signals had a noticeable dip in this position (Fig. 4b). Among all tested chromatin marks, H3K4me3, H3K9ac and H3K27ac distribute around TSS most similarly to PHD domains (Figs. 3d, 4b). In addition, these three marks co-localized strongly with each other (Fig. 4c, Suppl. Figure 4e, f), with PHD domain binding sites (Fig. 4d, Suppl. Figure 5a, b) and chromatin states bound by the PHD domains (Fig. 3i, Suppl. Figure 5c). In contrast, H4 acetylation marks, and particularly H4K16ac, co-localized poorly with PHD domains (Fig. 4d). Together, these observations are consistent with a role of H3K4me3, H3K9ac or H3K27ac, but not H4K16ac, as targets of clustered PHD domains in a cellular chromatin context.

Overall, PHD domains, full-length KMT2A and hCOMPASS-like subunits (WDR5 and RbBP5) co-localized with similar chromatin marks—H3K4me3, H3K9ac, H3K27ac and H3K23ac. However, H3K23ac overlapped better with full-length proteins than with PHD domains (Fig. 4d). Additionally, we investigated the intersections of the strongest binders (H3K4me1/3, H3K9ac, H3K23ac and H3K27ac). We observed that the correlation of PHDs and full-length proteins with the combination of H3K4me3/H3K9ac/H3K27ac and H3K9ac/K23ac/K27ac, respectively, was stronger than with single modifications (Fig. 4e-f). Taken together, the data suggest that the clustered PHD domains recognize multiple histone modifications. Biochemically, this is plausible, since each PHD domain may have separate methyl- and/or acetyl-binding sites.

To probe a possible role of H3K4me3, H3K9ac or K3K27ac in the recruitment of PHDs in vitro, we examined interactions of the purified GST-tagged proteins (Fig. 2a) with synthetic histone tail peptides (H3 unmodified, H3K4me3, H3K9acK27ac) in a peptide pull-down experiment (Fig. 4g). We observed that the PHD domains bound better to the H3K4me3 histone tail peptide than to the unmodified or the H3K9acK27ac doubly acetylated tail peptide (Fig. 4g). Thus, this experiment suggests a direct role of H3K4me3 in PHD domain recruitment. Using Far-Western, we showed that treatment of HeLa cells with valproic acid (VPA), a histone deacetylase (HDAC) inhibitor, strongly increased H3K27 acetylation, but had little effect on the binding of PHDs to mononucleosomes isolated from these cells (Suppl. Figure 5d). The findings suggest that the strong association of PHD domains with acetylated chromatin, seen in the gC&R data (Fig. 4d), could stem (in part) from indirect effects, such as increased histone tail peptide availability as a consequence of acetylation [36].

### A tandem of 2A13 PHDs with CXXC strongly improves promoter-specific recruitment

**Fig. 5:**
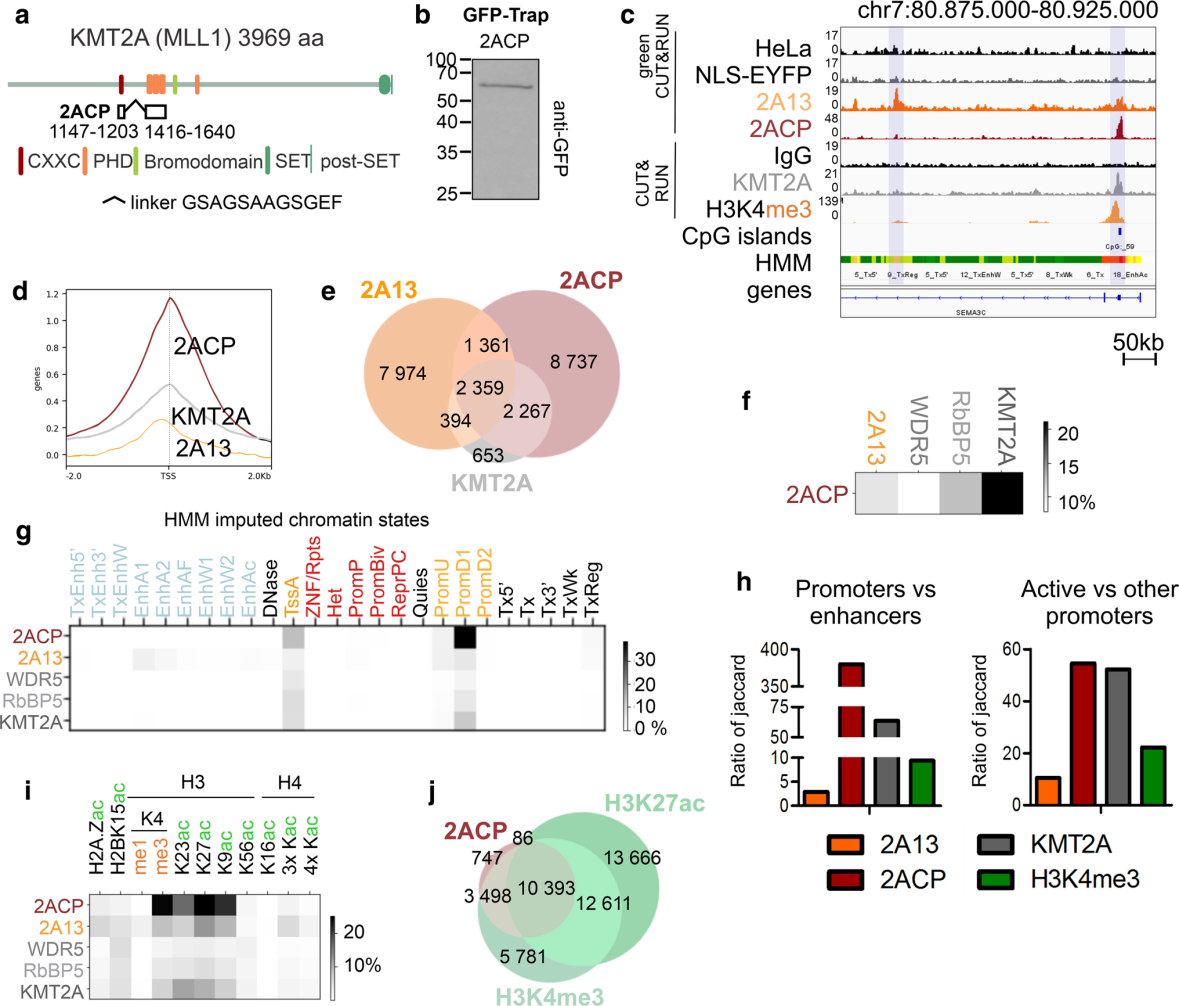
Tandem CXXC-2A13 of KMT2A protein improves promoter-specificity and overlap with full-length KMT2A in comparison with PHDs alone. **a** Scheme of the tandem CXXC-PHDs (2ACP) protein construct used for the generation of NLS-EYFP-2ACP stable HeLa S3 cell line by lentiviral transduction. **b** Western blot with anti-GFP antibody after GFP-Trap on NLS-EYFP-2ACP HeLa S3 stable cell lines. **c** IGV genome browser showing the distribution of reads within a representative gene (SEMA3C) generated by gC&R (2A13, 2ACP and negative controls HeLa, NLS-EYFP), CUT&RUN (KMT2A, H3K4me3 and negative control IgG), CpG islands (UCSC) and HMM chromatin states. **d** Heatmap plots around the TSS region (± 2 kb) and **e** Venn diagram comparing binding sites of full-length KMT2A, tandem 2ACP and clustered PHDs 2A13. **f** Jaccard analysis comparing the genome-wide peak distribution of tandem 2ACP, full-length KMT2A, hCOMPASS-like subunits (WDR5, RBbP5) and clustered PHDs 2A13. **g** Jaccard analysis comparing the genome-wide distribution of the peaks of 2ACP, 2A13, full-length KMT2A, RbBP5, and WDR5 with HMM imputed chromatin states. **j** Quantification of Jaccard ratios between loci enriched with indicated proteins (clustered PHDs 2A13, tandem CXXC-PHDs 2ACP, full-length KMT2A and H3K4me3). Left panel—the ratio of all promoters (active TSS—TssA, promoter downstream TSS—PromD1/2, promoter upstream TSS—PromU, poised promoters—PromP, bivalent promoters—PromBiv) and enhancers (active enhancer—EnhA1/2, active enhancer flank—EnhAF, weak enhancer—EnhW1/2, H3K27ac possible enhancer—EnhAc, transcribed and enhancer—TxEnh5’/3’, transcribed and weak enhancer—TxEnhW). Right panel—ratio of active promoters (TssA, PromD1, PromD2, PromU) and other promoters (PromP, PromBiv). **i** Jaccard analysis comparing the genome-wide distribution of the peaks of 2ACP, 2A13, full-length KMT2A, RbBP5, WDR5 and previously selected (Fig. 4a, d) chromatin marks. A darker signal indicates a more pronounced overlap. Enhancers are in light blue, promoters in yellow, poised or repressed states in red, and other regulatory elements in black. **j** Venn diagram comparing binding sites of tandem 2ACP and with regions enriched for the most correlated histone marks (H3K4me and H3K27ac)

Surprisingly, the specificity of clustered PHDs from promoter-specific KMT2A and enhancer-specific KMT2C-D was similar (Fig. 3i). In full-length KMT2A, but not KMT2C-D, the clustered PHD domains are adjacent to other chromatin-binding domains like the CXXC domain (binds CpG-rich DNA) or the bromodomain (binds acetylated histones). To probe domain cooperation and the effect on specificity, we aimed to generate stable HeLa cell lines featuring combinations of known chromatin-binding domains of the KMT2A-D proteins. Unfortunately, we did not achieve a stable cell line with a construct covering all domains, possibly due to their strong interactions with chromatin and resulting toxicity. Instead, we obtained stable cell lines with the very modest expression of artificial fusions of two clustered PHD domains from KMT2C (2C2P) or KMT2D (2D2P), a tandem of CXXC-PHD (2ACP) and cell lines with the stronger expression of the KMT2C-D SET domains (2CS, 2DS) (Fig. 5a, b, Suppl. Figure 6a-d). gC&R data for 2C2P, 2D2P did not indicate specific binding (Suppl. Figure 6e), possibly due to a not optimal choice of the linkers between clustered PHDs in 2C2P and 2D2P construct. Similarly, the gC&R data for the 2CS and 2DS cell lines showed poor chromatin affinity of the isolated SET domain (Suppl. Fig. 6e).

The 2ACP construct, a fusion of the CXXC and 2A13 PHD domains, recapitulated the KMT2A binding pattern much better than the 2A13 PHD domains alone (Fig. 5c-f). PHDs alone tended to be most highly enriched upstream to TSS (~ 150–200 bp) (Fig. 3d), whereas 2ACP and full-length KMT2A were most highly enriched directly at the TSS (Fig. 5d). 2ACP bound a CG-rich DNA motif, following the known properties of the CXXC domain (Suppl. Figure 6f). Compared to 2A13, 2ACP had a stronger preference for promoters over enhancers, even exceeding the preference of full-length KMT2A (Fig. 5g, h). For 2ACP, the most correlated chromatin marks were H3K4m3, H3K9ac, K23ac, and K27ac as for 2A13 , but the overlap was stronger (Fig. 5i, j). Taken together, these data show that the promoter-specificity of KMT2A is due to the interplay of the PHDs (active promoters and enhancers) and CXXC (active promoters) chromatin reader domains.

### PHDs bind chromatin regions involved in cancer-related pathways and are often mutated in cancer patients

**Fig. 6:**
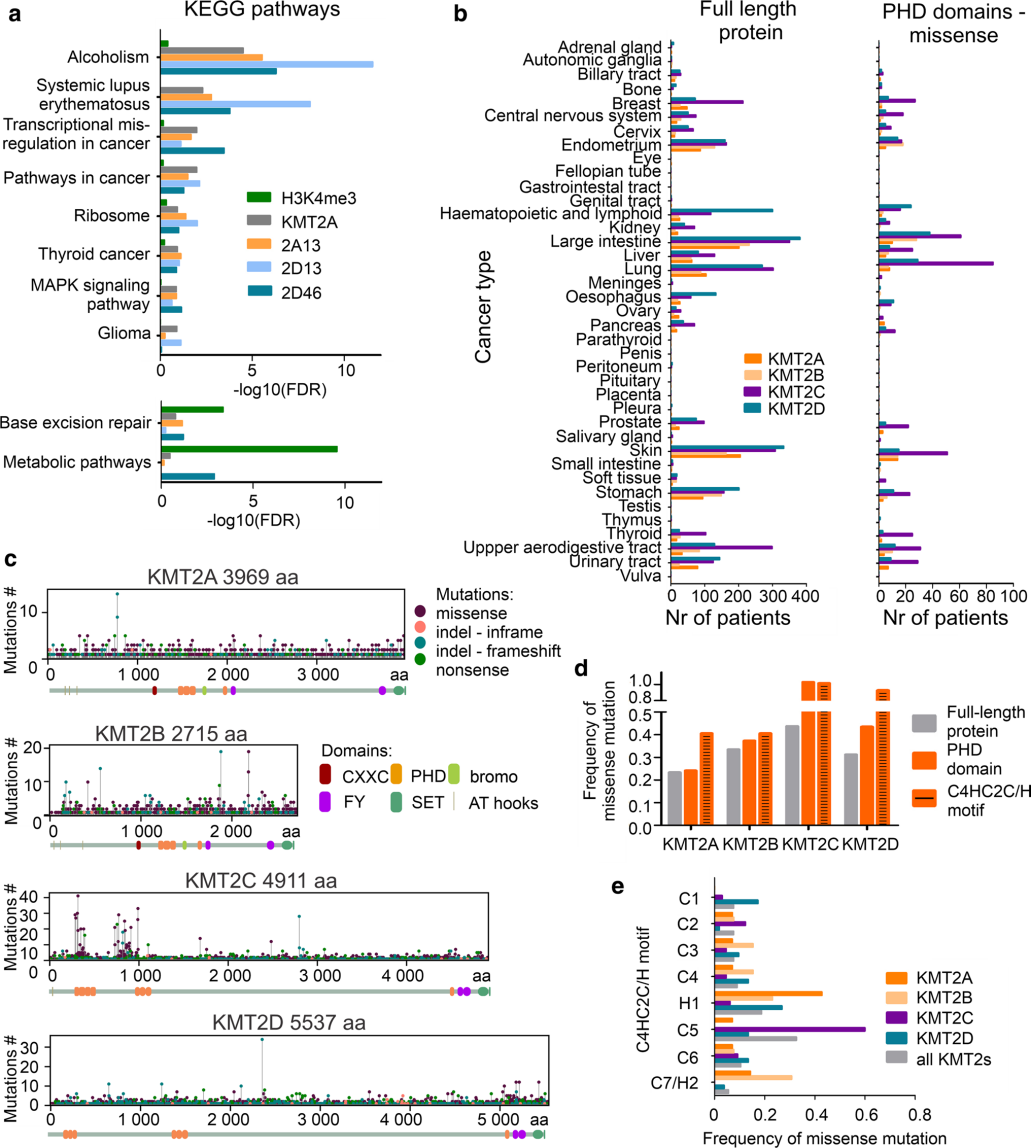
KMT2A-D PHDs bind loci involved in the cancer-related pathways. KMT2A-D PHDs, especially KMT2C, are mutated in cancer patients within crucial amino acids involved in the domain folding. **a** Top common hits of the KEGG pathway of the peaks identified for full-length KMT2A (gray) and clustered PHDs. H3K4me3 serves as a control showing the background of actively transcribed genes in HeLa S3 cells. FDR—false discovery rate. The top twelve hits for all proteins and GO biological processes are shown separately in Suppl. Figure 7a, b. 2C14 and 2C57 were not included in the analysis due to the low number of identified binding sites. **b-e** Data obtained from the COSMIC database [70]. **b** Distribution of mutations among cancer patients. Left panel—missense, nonsense and in-del mutations within full-length KMT2A-D. Right panel—missense mutations within PHDs of KMT2A-D. A comparison of missense within full-length KMT2A-D and missense within PHDs is shown in Suppl. Figure 8. **c** Distribution of missense (violet), in-frame (pink), frameshift (light blue), and nonsense (green) mutations within KMT2A-D full-length proteins. **d** The ratio between missense mutations and the number of residues within a particular protein region (full-length, PHD, C4HC2C/H motif). Data are collected separately for KMT2A-D. **e** The frequency of missense mutations within the Zn^2+^ binding motifs (Cys4HisCys2Cys/His, C4HC2C/H motif) of the PHDs. Gray: mutations in all KMT2A-D proteins, orange: KMT2A, light orange: KMT2B, violet: KMT2C, blue: KMT2D

With gC&R and C&R data, we performed KEGG and gene ontology (GO) analysis for the chromatin targets of NLS-EYFP-2A13, NLS-EYFP-2D13, NLS-EYFP-2D46 PHDs, and full-length KMT2A (Fig. 6a, Suppl. Figure 7a, b). Due to the small number of identified binding sites, we excluded 2C14 and 2C57 from this analysis. As a background control, we used H3K4me3 peaks, which showed the general active transcription in HeLa S3 cells. KEGG pathway analysis of bound genomic regions yielded similar results for all PHDs and full-length KMT2A protein (Fig. 6a, Suppl. Figure 7a). Pathways with the lowest false discovery rate (FDR), not observed in the general active transcription control (H3K4me3), were related to oncogenesis (e.g., “transcriptional misregulation in cancer” and “pathways in cancer”), following the known role of KMT2A-D and particularly of KMT2C as a tumor suppressor [37]. We observed the most significant associations with alcoholism and systemic lupus erythematosus, as previously observed for the HOXB1 transcription factor [38]. Results of the GO analysis of biological processes showed that most targets of clustered PHDs, but not H3K4me3, were associated with regulation of the mitotic cell cycle (Suppl. Figure 7b). In the case of 2D13, developmental processes were also prominent.

As KMT2A-D proteins are frequently mutated in cancer [10], we used the COSMIC database [39] to gain more insight into the role of PHD domains. According to the database, among the KMT2A-D proteins, KMT2C is the most frequently mutated (~ 4 050 cases of cancer patients), followed by KMT2D (~ 2 600 cases), KMT2A (~ 1 580 cases), and finally KMT2B (~ 1 250 cases). In addition, KMT2A is most frequently affected by translocations that result in mixed lineage leukemia (MLL). In contrast, point mutations in KMT2A-D proteins are most frequent in patients diagnosed with lung, liver, skin, or large intestine cancers (Fig. 6b). This pattern is similar when looking at mutations in full-length proteins or PHD domains only (Suppl. Figure 8).

Furthermore, our analysis of the COSMIC data for KMT2A-D genes showed that most of the mutations in cancer patients were found within the intrinsically disordered regions (IDRs), which make up most of the proteins (Fig. 6c). The only exception to this trend was KMT2C. Mutations in the gene encoding this protein are enriched within the clustered PHDs, particularly the first cluster 2C14, as previously observed [10]. Some of the mutations in KMT2A-D genes alter crucial amino acids of the PHDs (especially within KMT2A and KMT2D proteins), specifically Cys or His residues (within the Cys4HisCys2Cys/His motif) that are needed for chelating Zn^2+^ ions and therefore domain folding (Fig. 6d-e).

**Fig. 7:**
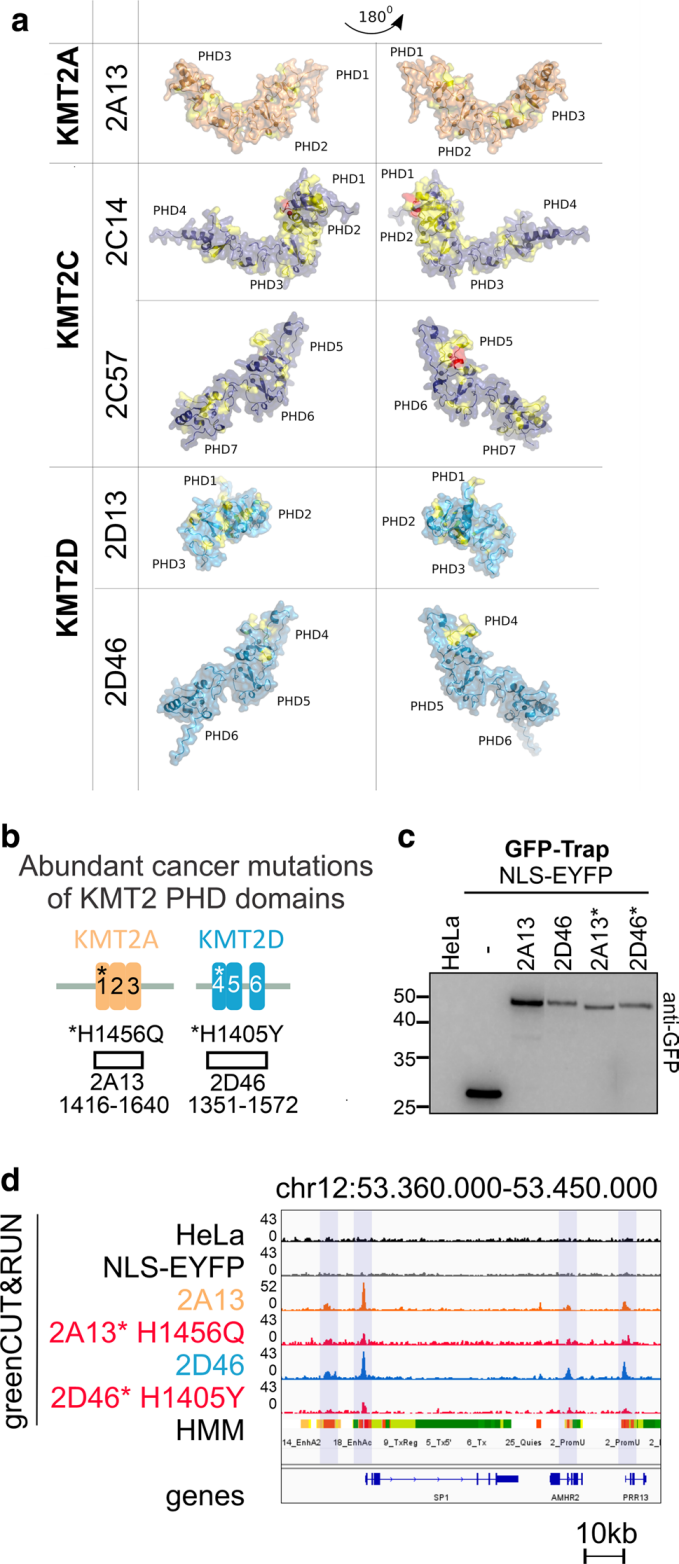
Mutations of PHD domains found in cancer patients impair chromatin reader properties. **a** Structure of PHDs predicted by Alphafold with COSMIC mutations mapped to the protein surface. Mutations found in 1–10 or more COSMIC patients are shown in yellow and red, respectively. **b** Scheme of the clustered PHDs of KMT2A-D proteins with mutations selected for experimental analysis, **c** Western blot with anti-GFP antibody after GFP-Trap of NLS-EYFP, 2A13, 2D46 and of their cancer mutation variants. **d** IGV genome browser snapshot illustrating impaired chromatin binding of the mutated PHD domains (2A13* H1456Q and 2D46* H1405Y) in comparison with the wild-type 2A13 and 2D46 domains

In order to map these mutations to the domain surface of KMT2A and KMT2C-D PHD, we generated AlphaFold-based models [40] and observed that these mutations tend to cluster spatially (Fig. 7a). To test the effect of most abundant cancer mutations on the chromatin-binding properties of PHDs by gC&R, we chose 2A13 and 2D46 PHDs. We based our choice on the the specific binding preferences of the wild-type domains (Fig. 3c), and the observation that mutations within these domains are enriched in the C4HC2C/H motif, especially H1 (Fig. 6e). Thus, we generated mutated HeLa cell lines bearing single missense mutations (His1456Glu 2A13*, His1405Tyr 2D46*), localized in the folding motif (Fig. 7b-c). In all cases, peaks were drastically reduced compared to wild-type controls, confirming the expectation that the cancer mutations cause loss-of-function and reduce or abolish target specificity on chromatin (Fig. 7d).

## Discussion

### GreenCUT&RUN as a tool to characterize chromatin readers

Chromatin reader domains are frequently studied in vitro using peptide pull-down and histone arrays with short and modified histone peptides [41–43]. These approaches are problematic because they do not take into account the chromatin context. Recently, synthetic nucleosomes with defined chromatin modifications have become commercially available as an alternative to synthetic histone tail peptides for research on chromatin interactions [44]. Experiments with such nucleosomes have demonstrated that some histone tail modifications affect chromatin reader binding indirectly, by modulating histone tail availability [36, 45]. The synthetic nucleosomes with defined posttranslational modifications have already been used for a so-called readerCUT&RUN assay [45]. Although this assay is more physiological than the assays with synthetic histone tail peptides, it still relies on reconstituted nucleosomes. ChIP-Seq and gC&R or C&R experiments are currently the preferred methods to characterize chromatin interactions in a physiological context. However, in our research, only gC&R is applicable for the clustered PHDs. Compared to the more traditional ChIP-Seq experiments, both gC&R and C&R experiments require fewer reads and benefit from a much better signal-to-noise ratio. We, therefore, believe that gC&R and C&R experiments could become useful tools to characterize chromatin readers.

### Clustered PHDs of KMT2A-D bind chromatin regions enriched in H3K4me3 and H3 acetylation

In this study, we observed in vitro that collaboration between the single modules of clustered PHDs in KMT2A-D strengthens their interactions with histones (Fig. 2e, f). We believe that KMT2A-D possess this unique organization of the PHD domains in triplets or even quartet clusters because of the need for stronger chromatin affinity. However, we cannot rule out that not investigated single domains are stronger binders, affecting the final readout.

**Fig. 8:**
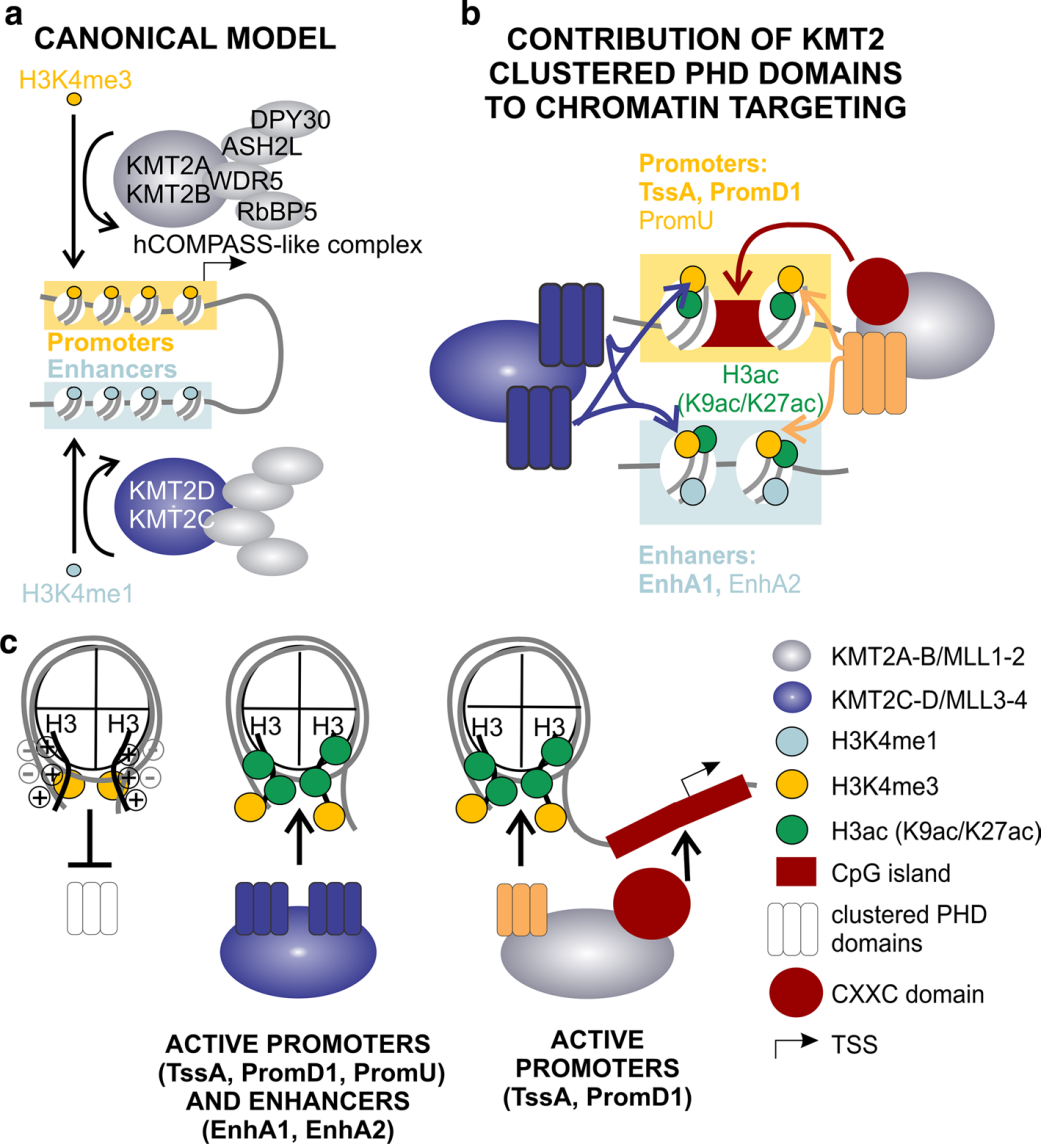
Involvement of PHDs in targeting of hCOMPASS-like complexes. **a** Canonically, KMT2A-B are involved in recognizing active promoters and catalyzing H3K4me3. In contrast, KMT2C-D are shown to target active enhancers and introduce H3K4me1. **b** We show that chromatin reader domains, such as clustered PHDs, are involved in targeting KMT2A-D proteins to active promoters (in particular around TSS (TssA) and within promoter PromD1) and to a lesser extent to active enhancers (EnhA1, EnhA2) rich in H3K4me3, H3K9ac and K3K27ac. The CXXC domain is present only within promoter-specific KMT2A and KMT2B, but not enhancer-specific KMT2C and KMT2D. It is highly attracted by non-modified CpG islands (any single modification repels this interaction), and enhances KMT2A-B promoter preference. **c** Scheme of nucleosome interactions between H3 histone tail, DNA and reading domains (PHDs and CXXC). Left panel—positively charged H3 tail interacts with dyad DNA, so that H3K4me3 is hidden and PHD domains are not bound. Middle panel—acetylated H3 tail (i.e., containing H3K9ac and H3K27ac) loses positive charge, so the H3K4me3 is available for the interaction with PHDs. Right panel—in the case of promoter-specific KMT2A, we observe cooperation of two reading domains—PHDs reading available H3K4me3 and CXXC reading unmodified CpG island within nucleosome free region (NFR) at TSS

In cells, we noticed a strong overlap of PHDs with multiple acetylation marks (Fig. 4d, Suppl. Figure 4c, d). These acetylation marks are all expected to be found in active chromatin, in line with the biological role of epigenetic memory proteins, like KMT2A-D (Fig. 8a). Among genomic elements of the same type, PHDs appear to discriminate chromatin states based on their acetylation levels. Preferentially bound promoter PromD1 is highly acetylated while unbound promoter PromD2 is not (Suppl. Figure 5c). Highly acetylated enhancer EnhA1 is preferred by PHDs over less acetylated enhancer EnhA2 (Suppl. Figure 5c). We also observed that clustered PHDs peaked more sharply around the TSS than single chromatin marks (Fig. 3d ~ 1 kb, Fig. 4b ~ 2–3 kb wide). This finding suggests that PHDs bind several chromatin marks simultaneously.

Our gC&R and C&R data show that H3K4me3, acetylation marks H3K9ac and H3K27ac and their intersections have the highest overlap score in the Jaccard analysis with PHDs (Fig. 4d-e). These marks are also distributed most similarly to PHDs around the TSS (Fig. 3d, Fig. 4b). Our data do not support the previously reported binding of singlet 2D6 to H4K16ac (Fig. 4a, b, d). This interaction was characterized using a synthetic peptide [31], which may not faithfully recapitulate the interactions with a full nucleosome.

H3K9ac and H3K27ac distinguish active from poised promoters and enhancers [46, 47]. Enrichment of the PHDs at these sites is therefore consistent with a preference of the PHD domains for active promoters and enhancers (Fig. 3i, Fig. 8b). However, in vitro peptide pull-down experiments indicate similar affinity of the PHD domains to unmodified and H3K9acH3K27ac histone peptide tails (Fig. 4g). Moreover, hyperacetylated mononucleosomes (isolated from VPA treated HeLa cells) have equal or at most minimally higher affinity to 2D46 compared to control non-treated (NT) mononucleosomes (Suppl. Figure 4d). Both experiments suggest that PHDs bind acetyl marks indirectly. Nucleosomes in cells have H3 tails in proximity to the entry and exit sites for the DNA dyad [48]. Therefore, our observations are compatible with the model that acetylation could decrease interactions with nucleosomal and linker DNA, thereby increasing H3K4me3 availability, and thus promoting binding of PHDs (Fig. 8c), as has been suggested for the PHDs of BPTF [36].

PHD domains of promoter-specific KMT2A and enhancer-specific KMT2C and KMT2D differ considerably (conservation 40–50%, Suppl. Figure 1b). Yet, all of them are recruited to H3K4me3 and H3 acetylation-rich active promoters and enhancers (Fig. 3i). The acetylation marks that overlap best with PHD domain binding sites, H3K9ac and H3K27ac, are known as both promoter and enhancer marks. By contrast, H3K4me3 is canonically associated with promoter regions. However, H3K4me3 was also shown to be present at strong enhancers [49]. In our data, H3K4me3 is found in ~ 23% and ~ 10% of the EnhA1 and EnhA2 active enhancer regions, respectively (Suppl. Figure 9a). By contrast, H3K4me3 decorates 76% TssA, 90% PromD1, 70% PromD2 and 61% PromU (Supp. Figure 9b). In general, 2A13 binds around 10% and 5% of EnhA1 and EnhA2, respectively (Suppl. Figure 9c). However, among the H3K4me3 decorated EnhA1 and EnhA2 enhancers, this overlap is several-fold larger, as 30% (1729 out of 5717) and 25% (791 out of 3135) are bound by 2A13, respectively (Suppl. Figure 9d). Together, these data confirm the observation that clustered PHDs recognize both active promoters and enhancers by targeting regions marked by H3K4me3 and H3ac (especially H3K9ac and H3K27ac) (Fig. 8c).

### The CXXC domain strongly enhances the promoter preference of KMT2A

The surprisingly similar binding preferences of PHD domains from promoter- and enhancer-specific KMT2A-D prompted us to focus on the overall architecture of these proteins. Interestingly, only promoter-specific KMT2A-B, but not enhancer-specific KMT2C-D, contain bromodomains and CXXC domains (Fig. 1a). Bromodomains typically bind acetylated lysine residues [50], associated with a permissive chromatin state [51]. They could therefore potentially help to target methyltransferases to active chromatin, thus facilitating positive transcriptional memory. Currently, there is no evidence that the bromodomains could contribute to the preference of KMT2A-B for promoters over enhancers. By contrast, KMT2A CXXC domains bind only at unmodified CpGs and are repelled by any single cytosine modification [12, 23]. Unmodified CpGs are enriched in CpG islands (CGIs) mostly within active promoters, but not elsewhere in the genome [24].

The gC&R data in this study show that an artificially fused tandem of CXXC and 2A13 strongly recapitulates the promoter-specific properties of full-length KMT2A (Fig. 5c-h). We also observed that 2A13 PHDs peak upstream of the TSS (~ 150-200nt), whereas 2ACP and full-length KMT2A peak directly at the TSS. We attribute the TSS upstream binding of 2A13 (and other PHDs) to the nucleosome-free region (NFR) directly at the TSS of the transcribed genes [52]. In contrast, 2ACP and full-length KMT2A can bind directly at the TSS because the CXXC domain binds CG-rich DNA directly and is not dependent on the presence of nucleosomes (Fig. 8c right panel, Suppl. Figure 6f). Additionally, the gC&R data indicate significantly higher promoter to enhancer preference for 2ACP than for 2A13 or even full-length KMT2A (Fig. 5g, h). Therefore, our findings strongly suggest that the presence of the CXXC domain in KMT2A-B, but not KMT2C-D, accounts for the observed stronger promoter preference of KMT2A-B compared to KMT2C-D (Fig. 8c). As KMT2C-D catalyze H3K4me1 [53, 54], present in both enhancers and promoters, we believe that their PHD domains may indeed target both chromatin states (active enhancers and promoters), not exclusively just the active enhancers (Fig. 8b, c).

### Chromatin targeting by other hCOMPASS-like subunits

KMT2A-D proteins are recruited to their chromatin targets not only by their reading domains. Their localization can also be influenced by their association with other chromatin reader subunits of hCOMPASS-like complexes. For WDR5, a preference for methylated H3K4, particularly dimethylated H3K4 [14, 55, 56] with modulation by methylation of H3R2 [19] and serotonylation of H3Q5, was demonstrated biochemically [57]. For ASH2L, strong evidence for DNA binding was shown [18, 58]. Menin, a component of hCOMPASS-like complexes with KMT2A-B catalytic core was observed to bind a plethora of transcription factors [59]. Despite all these influences on KMT2A-D targeting, our data show that the 2A13 triple PHD domain of KMT2A and the CXXC-2A13 tandem bind to similar targets as full-length KMT2A in the hCOMPASS-like complex, suggesting that these domains make an important contribution to the protein targeting.

### Clustered PHDs in KMT2A-D proteins are conserved and developmentally required in animals

The clustered PHD domains are among the most highly conserved regions of KMT2A-D proteins (Fig. 1b). They occur in vertebrate and invertebrate KMT2A-D orthologues. The *Drosophila melanogaster* orthologue of KMT2A-B, Trx, has one PHD triplet. The *Drosophila* orthologue of KMT2C-D, Trr, lacks clustered PHDs but interacts with a separate protein known as lost PHDs (*Lpt* or *Cmi*) that contains two clusters of PHDs (a quartet and a triplet). In Drosophila, ablation of *Cmi* results in larval lethality [60], at least in part due to defects in pattern formation [61]. This observation highlights the role of clustered PHDs in organism development. To our knowledge, triplets and quartets of PHD domains are unique to the animal KMT2A-D proteins and not found elsewhere.

### The clustered PHDs in KMT2A-D proteins are frequently mutated in cancer

Missense and nonsense mutations, as well as small deletions and insertions, are frequent in KMT2A-D and some of them map to the PHDs (Figs. 6b, c, 7a). Highly significant enrichment is seen for the frequently mutated KMT2C, as observed previously [11]. The distribution of mutations to many sites, rather than a few selected sites, and the enrichment of alterations in conserved residues of the PHDs suggest that the mutations cause a loss-of-function and change-of-function, respectively [62]. The accumulation of alterations in conserved residues of the PHDs suggests that PHD dysfunction is an important contributor to KMT2A-D-driven malignancies. This hypothesis is supported by our observation of frequent mutations within amino acids required for the folding of the PHD domains (Fig. 6d-e) and by the demonstration that the mutations disrupt domain targeting (Fig. 7d). However, KMT2A alterations in leukemia, particularly mixed lineage leukemia, are not small local changes, but chromosomal translocations generating novel fusion proteins. Importantly, translocation breakpoints cluster into a major Breakpoint Cluster Region (BCR) with most breakpoints in the core region from exon 9 to 11 [63, 64], and a minor breakpoint cluster region from exons 19 to 26 [65]. As the coding region for clustered PHDs of KMT2A (2A13) starts in exon 11, 2A13 is either completely absent or only present in a truncated form within the oncogenic fusion proteins. Thus, we conclude that the biology of PHDs is relevant for KMT2A-D loss-of-function malignancies, but not for translocation-driven KMT2A gain-of-function leukemia.

In conclusion, we have shown that clustered PHD domains direct KMT2A and KMT2C-D to a subset of active promoters (in particular close to TSS) and to a lesser extent to active enhancers of cancer-related pathways (Fig. 8b, c). PHDs bind the chromatin loci cooperatively by recognizing regions enriched in H3K4me3 and H3ac (H3K9ac and H3K27ac). In our experiments, we have shown that mutations in the PHD domains found in cancer patients disrupt the targeting of the isolated domains. These mutations are likely selected in cancer because they also disrupt or at least negatively impact targeting of the entire hCOMPASS-like complexes. Hence, our research suggests a role of the PHD domains of KMT2A-D proteins in the maintenance of positive epigenetic memory.

## Materials and methods

### Cloning and overexpression of PHD domains

*E. coli* codon-optimized synthetic genes (GeneArt, ThermoScientific; Table S1) encoding the PHD domains of human KMT2A (UniProt: Q03164, 2A3, aa 1565–1631; 2A13, aa 1416–1640), KMT2B (UniProt: Q9UMN6, 2B13, aa 1182–1414), KMT2C (UniProt: Q8NEZ4, 2C14, aa 266–532; 2C7, aa 1083–1143; 2Ce7, aa 1055–1144; 2C67, aa 1008–1144; 2C57, aa 934–1149) and KMT2D (UniProt: O14686, 2D13, aa 156–341; 2D6, aa 1503–1561; 2De6, aa 1475–1564; 2D46, aa 1351–1572) were cloned using BamHI and XhoI restriction sites into pGEX-P6-2 plasmids encoding an N-terminal GST-tag (Fig. 2b). For GST overexpression, an empty pGEX-P6-2 plasmid was used. In addition, human codon-optimized synthetic genes (GeneArt, ThermoScientific; Table S1) encoding the clustered PHD domains of KMT2A (UniProt: Q03164, 2A13, aa 1565–1631), KMT2C (UniProt: Q8NEZ4, 2C14, aa 266–532; 2C57, aa 934–1149) and KMT2D (UniProt: O14686, 2D13, aa 156–341; 2D46, aa 1351–1572) were cloned using the SLIC method [66, 67], first to pEYFP plasmids and then into a customized pLJM1-EGF1-based plasmid encoding N-terminal EYFP for the generation of a stable cell line by lentiviral transduction. Briefly, NLS-EYFP-PHD fusion gene fragments were amplified with the primers specified in Table S2 using Q5 DNA Polymerase. Plasmid pLJM1 was linearised using AgeI and EcoRI restriction enzymes. DNA fragments were incubated for 30 min with T4 DNA polymerase at 22 °C to generate overhangs. The reaction was stopped by the addition of dCTP to a 2 mM final concentration. Inserts and vectors were mixed in a 1:1 molar ratio and incubated for 30 min at 37 °C before being transformed into NEB stable *E. coli* competent cells.

Other human codon-optimized synthetic genes (GeneArt, ThermoScientific; Table S1) encoding tandem reading domains KMT2A (UniProt: Q03164, 2ACP, aa 1147–1203 and 1416–1640), KMT2C (UniProt: Q8NEZ4, 2C2P, aa 266–532 and 934–1149), KMT2D (UniProt: O14686, 2D2P, aa 156–341 and 1351–1572) fused with the artificial linker GSAGSAAGSGEF, SET domains of KMT2C (UniProt: Q8NEZ4, 2DS, aa 4751–4911) and KMT2D (UniProt: O14686, 2DS, aa 5371–55,537) and cancer-related mutations in KMT2A PHDs (UniProt: Q03164, 2A13* H1456Q, aa 1416–1640), KMT2D (UniProt: O14686, 2D46* H1405Y, aa 1351–1572) were cloned using BamHI and XhoI into a customized pLJM1-EGF1-based plasmid encoding N-terminal EYFP for the generation of a stable cell line by lentiviral transduction.

All protein overexpression was performed in *E. coli* BL21(DE3) pLys cells. The cells were grown in LB media supplemented with 100 ng/ml ampicillin at 37 °C until OD_600_ reached 0.6/cm–0.8/cm. Cells were then transferred to 4 °C for 30 min. After induction with 1 mM IPTG and in the presence of 150 µM ZnSO_4_, proteins were expressed overnight at 22 °C. The cells were then harvested and stored at -80 °C in pellets from 1L of culture batches.

### Protein purification

Frozen cells overexpressing GST or GST-PHDs were resuspended in 40 ml ice-cold lysis buffer (50 mM Tris–HCl pH 7.5, 400 mM NaCl, 5% glycerol, 150 µM ZnSO_4_, 1 mM DTT) supplemented with 0.25 mM PMSF. After sonication, the lysate was cleared by ultracentrifugation for 30 min. Next, the supernatant was applied to Glutathione Sepharose™ 4B beads (GE Healthcare) equilibrated with lysis buffer. After 2 h of incubation, the beads were extensively washed with washing buffer (50 mM Tris–HCl pH 8.0, 400 mM NaCl, 5% glycerol, 150 µM ZnSO_4_, 1 mM DTT). Protein was eluted by incubating the beads with elution buffer (200 mM Tris–HCl pH 8.0, 150 mM NaCl, 5% glycerol, 20 mM reduced L-glutathione, 150 µM ZnSO_4_, 1 mM DTT) for 15 min. The purified protein was concentrated using Amicon Ultra centrifugal filters with 10 kDa cutoff (Millipore) and dialyzed overnight against storage buffer (50 mM Tris pH 8.0, 150 mM NaCl, 150 μM ZnSO_4_, 1 mM DTT, 50% glycerol). Protein concentrations were determined spectrophotometrically (Nanodrop, Thermo Fisher Scientific). Aliquoted samples were frozen in liquid nitrogen and stored at -80 °C.

### Isolation of mononucleosomes

Mononucleosomes were isolated from HeLa S3 cells. A pellet of 20 million HeLa S3 cells was resuspended in 5 ml of ice-cold TM2 buffer (10 mM Tris pH 7.5, 2 mM MgCl_2_, 0.5 mM PMSF). Next, NP-40 was added to a final concentration of 0.6%. After incubation on ice for 5 min, the pellet was washed with TM2 buffer, resuspended in 400 μl of TM2 buffer containing micrococcal nuclease (New England Biolabs) and 1 mM CaCl_2_, and incubated in a Thermomixer for 10 min at 37 °C. Subsequently, 2 mM EGTA, 300 mM NaCl, and 0.1% Triton X-100 were added, and the mixture was transferred into Bioruptor tubes (Diagenode). After sonication for five cycles of 30 s ON/OFF at 4 °C (Bioruptor Pico, Diagenode), the mixture was centrifuged (13 000xg) for 10 min at 4 °C, and the supernatant was transferred to a fresh tube. For quality control, DNA was isolated from a small aliquot (PCR clean up, A&A Biotechnology) and checked on 1.5% agarose gel for DNA length (~ 150 nt). Finally, mononucleosomes were divided into aliquots, fast-frozen with liquid nitrogen, and stored at -80 °C.

### Human cell lines

HeLa S3 cells, HeLa S3-derived stable cell lines, and HEK293T cells were cultured in Dulbecco’s Modified Eagle Medium (DMEM, Biowest) supplemented with 10% fetal bovine serum (FBS, Biowest) and penicillin–streptomycin solution (Biowest) at 37 °C in a humidified 5% CO_2_ atmosphere. Cells were passaged using trypsin and stored in 5% DMSO and DMEM containing 10% FBS and antibiotics at -80 °C.

### Generation of stable cell lines

Stable HeLa S3 cell lines expressing NLS-EYFP-tagged proteins were generated using lentiviral transduction. Lentivirus stocks were prepared using plasmids pMD2.G and psPAX2, and a pLJM1-based transfer plasmid, by standard transfection with PEI (Sigma-Aldrich #408,727). HEK293T cells were grown in a 15 cm dish following transfection. Upon reaching ~ 90% cell confluency, the medium was aspirated and replaced with fresh medium without antibiotics. Transfer plasmid pLJM1 and plasmids psPAX2 and pMD2.G were mixed in a 4:2:1 ratio in Opti-MEM (20 µg, final volume 0.7 ml). Next, PEI in Opti-MEM was added (60 µg in a total volume of 0.7 ml). The transfection mixture was briefly vortexed, incubated for 20 min at room temperature, and slowly added to the cell culture. 17 h post-transfection, the medium was exchanged for fresh DMEM supplemented with 25 mM HEPES, 4 mM caffeine, and 1 mM sodium butyrate. The medium was harvested 48 h post-transfection and centrifuged for 3 min at 3 000 g. The supernatant was then filtered through a 0.45 µm PES filter, aliquoted, and stored at -80 °C.

To generate stable cell lines, 0.1 million HeLa S3 cells were seeded per well in a 6-well plate 24 h before infection. Infection was carried out for 72 h. At every 24 h, media was exchanged for a new virus aliquot and fresh media in a ratio of 2.5:0.5, supplemented with DEAE-dextran (6 µg/ml). The negative control NLS-EYFP HeLa S3 stable cell line was infected once after 24 h. 72 h after the first virus addition, the cells were transferred to a 10 cm plate and maintained in media supplemented with 2 µg/ml puromycin. Non-infected parental HeLa S3 cells were also treated with puromycin to confirm the successful selection.

### Flow cytometry and cell sorting

After successful puromycin selection, stable cell lines were sorted using a BD FACSAriaII (Becton Dickinson), in collaboration with the Microscopy and Cytometry Facility in IIMCB. Briefly, parental and stable HeLa S3 cell lines were washed with PBS, harvested with trypsin–EDTA, washed twice with PBS, resuspended in PBS buffer and transferred to 5 ml FACS tubes. Next, cells expressing EYFP above background levels of the parental HeLa S3 cell line were sorted out and transferred back to the culture plate. Sorted cells were cultured in standard conditions for at least 2 weeks to reach confluency, re-analyzed using BD FACSAria II cell sorter and used for the gC&R experiments. To detect the EYFP fluorescence signal in the cells blue laser 488 nm and FITC emission filter (530/30) were used. Finally, the cells were stored in 5% DMSO and DMEM containing 10% FBS and antibiotics at -80 °C. Flow cytometry data were plotted and analyzed using FlowJo software (Becton Dickinson).

### GST pull-down, peptide pull-down and GFP-trap

For GST pull-down, purified GST-tagged PHD domains (1 μM) were incubated with 30 μg of isolated mononucleosomes in 500 μl of pull-down buffer (10 mM Tris pH 7.5, 150 mM NaCl, 0.5% Triton X-100, 1 mM DTT, 150 μM ZnSO_4_, 1 mM PMSF, 1xEDTA-free protease inhibitors [Roche]) overnight on a rotating wheel at 4 °C. 10% of the mixtures were put aside as input. Next, 40 μl of Glutathione Sepharose™ 4B beads (GE Healthcare) were washed three times with pull-down buffer and added to the sample containing the PHD mononucleosome complexes. The mixtures were then incubated for 2 h on a rotating wheel at 4 °C. Following this, the supernatants were collected as flow-through and the beads were washed three times with washing buffer (10 mM Tris pH 7.5, 150 mM NaCl, 0.5% Triton X-100). Finally, the beads were incubated at 95 °C with SDS-loading buffer (50 mM Tris pH 6.8, 2% SDS, 5% β-mercaptoethanol, 10% glycerol, bromophenol blue) for 10 min for Western blot.

For peptide pull-down, first 25 μl Dynabeads™ MyOne™ Streptavidin C1 were incubated with 250 pmols of selected biotinylated peptides (GeneScript, Supplementary Table S3) in 100 μl of peptide binding buffer (50 mM Tris pH 8.0, 300 mM NaCl, 0.1% NP-40, 1 mM PMSF) for 30 min on a rotating wheel at room temperature. Next, resins were washed three times and incubated with GST-tagged PHD domains (25 pmol) in 300 μl protein binding buffer (50 mM Tris pH 8.0, 150 mM NaCl, 0.1% NP-40, 0.5% BSA, 1 mM DTT, 150 μM ZnSO4, 1 mM PMSF) for 4 h on a rotating wheel at 4 °C. 1% of the mixtures were put aside as input. Following this, the beads were washed three times with the protein binding buffer. Finally, the beads were boiled at 95 °C with SDS-loading buffer (50 mM Tris pH 6.8, 2% SDS, 5% β-mercaptoethanol, 10% glycerol, bromophenol blue) for 10 min for Western blot.

For GFP-Trap, 10 cm plates were seeded with the selected NLS-EYFP-tagged stable HeLa S3 cell line. When the plate reached full confluency (~ 10^7^ cells), the cells were washed with ice-cold PBS and scraped from the plate. Next, cells were washed two more times with ice-cold PBS and lysed with RIPA buffer lacking EDTA (10 mM Tris 7.5, 150 mM NaCl, 0.1% SDS, 1% Triton X-100, 1% deoxycholate), supplemented with DNaseI, 2.5 mM MgCl2, 1 mM PMSF and EDTA-free protease inhibitor cocktail (Roche) for 30 min on ice. The lysate was centrifuged at 20 000 g for 10 min at 4 °C, diluted with the dilution buffer (10 mM Tris pH 7.5, 150 mM NaCl, 1 mM PMSF) and mixed with 10 μl equilibrated GFT-Trap magnetic agarose beads (Chromotek). The lysate was incubated with the resins on the rotating wheel for 1 h at 4 °C. Finally, the flow through was discarded, and the resins were washed three times with the dilution buffer, resuspended in SDS-loading buffer (50 mM Tris pH 6.8, 2% SDS, 5% β-mercaptoethanol, 10% glycerol, bromophenol blue) and boiled for 5 min at 95 °C.

### SDS-PAGE, western blots, and Far-western blots

Proteins were diluted in SDS-loading buffer and heated at 95 °C for 5 min. The samples were then run on Tris/glycine/SDS–polyacrylamide gel (the 12% and 18% separating gel for GST-tagged PHDs and histone proteins, respectively). For the Western blots, proteins were wet-transferred onto a nitrocellulose membrane (100 V, 1 h, 4 °C). The membrane was blocked with 5% non-fat milk and incubated with a specific primary antibody for rabbit anti-histone H3 (Cell Signaling Technologies) or goat anti-GFP (R&D System) overnight at 4 °C. Next day, the membrane was washed three times with PBS-T (PBS with 0.1% Tween20), incubated at room temperature with anti-rabbit or anti-goat HRP-conjugated secondary antibodies (Sigma) for 1 h and washed three times with PBS-T. For detection of GST-tagged proteins, after blocking with 5% non-fat milk, the membrane was incubated at room temperature with an anti-GST HRP-conjugated primary antibody (Abcam) for 1 h and washed three times with PBS-T.

For Far-Western blotting, 5 μg of mononucleosomes were resolved using an 18% SDS-PAGE gel, wet-transferred onto a nitrocellulose membrane (1 h, 100 V, 4 °C), and blocked for 1 h at room temperature or overnight at 4 °C with 5% non-fat milk. The membranes were then washed three times with TBS-T and once with interaction buffer (20 mM HEPES 7.5, 100 mM KCl, 10% glycerol, 1 mM DTT, 150 μM ZnSO_4_). A subsequent incubation with 100 nM PHD domains in the interaction buffer for 1 h at room temperature was performed. The membranes were again washed three times with TBS-T and incubated with an anti-GST HRP-conjugated antibody (Abcam) for 1 h at room temperature. Finally, three washes times with TBS-T were done.

Immunodetection was performed using self-made ECL (0.2 mM coumaric acid, 1.25 mM luminol, 100 mM Tris 8.5, 0.03% H_2_O_2_) or Femto ECL (ThermoScientific). The chemiluminescent signals were scanned from the membranes using a BioRad documentation system. All antibodies used are listed in Table S4**.**

### Standard CUT&RUN (C&R) and greenCUT&RUN (gC&R)

For standard C&R [68], the protocol published by Janssens and Henikoff (10.17504/ protocols.io.zcpf2vn) was used. For gC&R, the protocol described by Nizamuddin and colleagues [69], with minor modifications, was used. Briefly, in both protocols, 0.5 million HeLa S3 cells were collected by trypsinisation and washed twice with wash buffer (20 mM HEPES–KOH pH 7.5, 150 mM NaCl, 0.5 mM spermidine and EDTA-free complete protease inhibitor cocktail). In parallel, BioMag®Plus Concanavalin A (ConA) magnetic beads (10 μl per 0.5 million HeLa S3 cells) were incubated twice with binding buffer (20 mM HEPES–KOH pH 7.5, 10 mM KCl, 1 mM CaCl_2_ and 1 mM MnCl_2_). The washed cells were then mixed with the conA beads and incubated on a vortexer for 8 min. All steps up to this point were performed at room temperature to minimize cellular stress and DNA fragmentation.

For C&R, conA immobilized cells were incubated with 100 μl antibody buffer (20 mM HEPES–KOH pH 7.5, 150 mM NaCl, 0.5 mM spermidine and EDTA-free protease inhibitor cocktail, 0.01% digitonin, 2 mM EDTA) supplemented with the antibody overnight on a rotating wheel at 4 °C. All antibodies used are listed in Table S4**.** Following overnight incubation, the cells were washed twice with ice-cold digitonin buffer and incubated on a nutator for 1 h with 50 μl digitonin buffer containing 1.5 μl C&R MNase (Cell Signaling Technologies). For gC&R, conA immobilized cells were incubated for 4 min at room temperature with antibody buffer and then washed twice with digitonin buffer. Next, the immobilized cells were incubated in 100 μl digitonin buffer with 0.4 ng of the fusion protein consisting of anti-GFP nanobody-MNase (a kind gift from Marc Timmers) for 1 h at 4 °C on a rotating wheel.

In both protocols, cells were then washed two times with ice-cold digitonin buffer, resuspended in 100 μl digitonin buffer, and kept on a wet-ice bath for at least 5 min. Next, MNase digestion was activated by adding CaCl_2_ to a 2 mM final concentration. After 30 min, digestion was stopped by adding 100 μl 2 × STOP buffer (340 mM NaCl, 20 mM EDTA, 10 mM EGTA, 0.01% digitonin, 100 μg/ml of RNase A [ThermoScientific]). Next, the mixtures were incubated in a thermoblock at 37 °C to release chromatin-MNase complexes. Supernatants (200 μl) were supplemented with 2 μl of 10% (w/v) SDS and 2.5 μl proteinase K (20 mg/ml; ThermoScientific) and incubated for 1 h at 50 °C in a thermoblock. DNA was extracted using a ChIP DNA Clean & Concentrator kit (ZymoResearch). For background correction in the C&R experiments, rabbit normal IgG (Sigma-Aldrich) was used, and in the gC&R experiments, the parental HeLa S3 and NLS-EYFP negative control cell lines were used.

### Library preparation

For library preparation, a NEBNext II Ultra DNA Prep Kit was used according to Liu’s protocol (10.17504/protocols.io.bagaibse). Briefly, DNA isolated after C&R or gC&R was mixed with END Prep Enzyme Mix and Reaction Buffer and incubated in a thermocycler at 20 °C for 30 min, and at 50 °C for 1 h. Next, NEB Next Adaptor (25 × diluted, 0.6 μM) was ligated using Ligation Master Mix and Ligation Enhancer for 15 min at 20 °C, followed by hairpin cleavage by USER enzyme for 15 min at 37 °C. For the cleaning of adaptor-ligated DNA, left-side selection using 1.7 × AMPure XP beads (Beckman Coulter) was used. The purified DNA was mixed with Q5 MasterMix, Universal PCR primer, and appropriate index primer (NEB Single Index, NEBNext® Multiplex Oligos for Illumina), and used for PCR in a thermocycler (BioRad, Eppendorf). Finally, the amplified DNA libraries were cleaned with AMPure XP beads (first 0.55 × right-sided, then left-sided in total of 1.15x). The quality of libraries was verified using TapeStation (Perlan, Agilent).

### NGS sequencing and data analysis

The concentration of the libraries was measured by qPCR using a Kapa Library Quantification kit (Kapa Biosciences, KK4824), according to the manufacturer’s protocol. Paired-end (2 × 100 nt) sequencing (10 MR histone modifications, 30 MR full-length hCOMPASS-like subunits and gC&R) was performed on an Illumina NovaSeq 6000 instrument, using a NovaSeq 6000 S1 Reagent Kit (200 cycles, Illumina) with the addition of 0.5% control library Phix (Illumina).

The analysis of sequencing data was performed using the Galaxy server [70]. Original fastq files were trimmed using both the TrimGalore! and FastQTrimmer algorithms. The quality of the reads was confirmed by FastQC. Next, the paired-end reads were aligned to the human genome hg38 using Bowtie2 [71], filtered for minimum MAPQ20 and against a blacklist, and deduplicated. Peak calling was done using MACS2 [72, 73]. Heatmaps were created using plotHeatmap from deeptools in Galaxy [74]. Reads were first analyzed in IGV [75], then peaks genome-wide using KEGG [76] and GO [77] analysis on the Cistrome-GO webserver [78]. For the comparison of the bed files with HMM imputed data or histone marks, the standard Jaccard index [79], a measure of interval overlap from bedtools [80], was used. Alternatively, the overlap was visualized using Venn diagram generated using Intervene [81].

### Conservation analysis (Shannon analysis)

For sequence conservation analysis, UniProt [82] was queried for proteins annotated as KMT2A, KMT2B, KMT2C, or KMT2D. The program cd-hit [83] was used to cluster sequences at 80% sequence similarity. Fusion proteins or truncated proteins (any proteins with a length less than 90% of the length of the reference human protein) were excluded from the analysis. To avoid bias from multiple annotated splice variants, only one protein per paralogue and species was retained. Proteins were aligned using Clustal Omega [84]. As in sequence logos, sequence conservation was defined as the difference between the maximally possible and actual Shannon entropy in bits (log2(20) + Σp_i_log2p_i_, where p_i_ is the frequency of amino acid “i” in a given position). As scores were needed concerning reference to the human protein and not to a gapped alignment, BALCONY was not used directly, and the calculation of Shannon scores was re-implemented in Python.

### Structural analysis based on AlphaFold structure predictions

For structural analysis of clustered PHD domains (2A13, 2C14, 2C57, 2D13 and 2D46), AlphaFold prediction results were used [40]. Based on the COSMIC data, Pymol and Chimera software was used for the labeling of the mutated sites.

### COSMIC data analysis

The data for the analysis of the distribution of mutations throughout the length of the KMT2A-D proteins and in the patient population were obtained from the Catalogue of Somatic Mutations in Cancer (COSMIC) release v.92 [39]. The COSMIC Mutation Data (Genome Screens) dataset containing data on mutations found in whole genome sequencing (WGS) analysis was downloaded from the database as a filtered file to comprise the changes found in the KMT2A–D genes in all the malignancies described in COSMIC. The data from targeted screens (non-WGS) were not used in the distribution analysis of mutations as too many data points (~ 92%) are missing concerning the type and location of mutations for the non-WGS dataset to be informative.

For the analysis of the distribution of mutations among patients, the COSMIC Sample Features non-filtered dataset was downloaded to calculate the number of patients diagnosed with a given malignancy and to assign the mutations described in the Mutation Data file to individuals. The data points from the WGS Mutation Data dataset with ‘Mutation description’ assigned as ‘Unknown’ (33% of the whole dataset) were removed from the analysis. Finally, the data were filtered for the position within the reading domain.

### Supplementary Information

Below is the link to the electronic supplementary material.

Supplementary file1 (PDF 1826 KB)

## Data Availability

All raw and processed sequencing green/CUT&RUN data generated in this study have been submitted to the NCBI Gene Expression Omnibus (GEO; https://www.ncbi.nlm.nih.gov/geo/) under accession number: GSE185921.
